# 2-(4-Bromo­phen­yl)-1-ethyl-1*H*-1,3-benzodiazole

**DOI:** 10.1107/S1600536811001097

**Published:** 2011-01-15

**Authors:** Su-Lan Dong, Xiao-Chun Cheng

**Affiliations:** a College of Life Science and Chemical Engineering, Huaiyin Institute of Technology, Huaiyin 223003, Jiangsu, People’s Republic of China

## Abstract

In the title compound, C_15_H_13_BrN_2_, the benzimidazole group is almost planar, as indicated by the dihedral angle of 2.6 (3)° between the best planes through the benzene and imidazole rings. The best plane through the attached benzene makes an angle of 44.5 (2)° with the best plane through the benzimidazole system. C—H⋯π inter­actions are observed in the crystal structure.

## Related literature

For the synthesis, see: Kakimoto *et al.* (2008 ▶). For bond-length data, see: Allen *et al.* (1987 ▶).
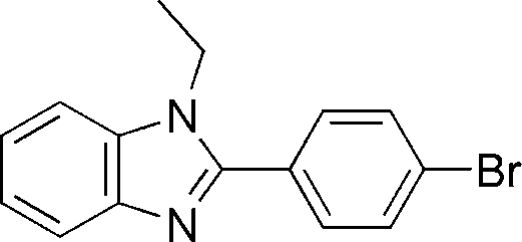


## Experimental

### 

#### Crystal data

C_15_H_13_BrN_2_
*M* *_r_* = 301.18
Triclinic, 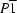

*a* = 9.0780 (18) Å
*b* = 9.1480 (18) Å
*c* = 9.2750 (19) Å
α = 76.72 (3)°
β = 78.44 (3)°
γ = 61.05 (3)°
*V* = 652.4 (2) Å^3^
*Z* = 2
Mo *K*α radiation
μ = 3.13 mm^−1^
*T* = 293 K
0.20 × 0.10 × 0.10 mm

#### Data collection

Enraf–Nonius CAD-4 diffractometer
Absorption correction: ψ scan (North *et al.*, 1968 ▶) *T*_min_ = 0.573, *T*_max_ = 0.745
2550 measured reflections
2388 independent reflections
1322 reflections with *I* > 2σ(*I*)
*R*_int_ = 0.068
3 standard reflections every 200 reflections  intensity decay: 1%

#### Refinement

*R*[*F* ^2^ > 2σ(*F* ^2^)] = 0.050
*wR*(*F* ^2^) = 0.111
*S* = 1.00
2388 reflections
163 parameters
H-atom parameters constrained
Δρ_max_ = 0.28 e Å^−3^
Δρ_min_ = −0.32 e Å^−3^

### 

Data collection: *CAD-4 Software* (Enraf–Nonius, 1985 ▶); cell refinement: *CAD-4 Software*; data reduction: *XCAD4* (Harms & Wocadlo, 1995 ▶); program(s) used to solve structure: *SHELXS97* (Sheldrick, 2008 ▶); program(s) used to refine structure: *SHELXL97* (Sheldrick, 2008 ▶); molecular graphics: *SHELXTL* (Sheldrick, 2008 ▶); software used to prepare material for publication: *SHELXTL*.

## Supplementary Material

Crystal structure: contains datablocks I, global. DOI: 10.1107/S1600536811001097/vm2072sup1.cif

Structure factors: contains datablocks I. DOI: 10.1107/S1600536811001097/vm2072Isup2.hkl

Additional supplementary materials:  crystallographic information; 3D view; checkCIF report

## Figures and Tables

**Table 1:** Hydrogen-bond geometry (Å, °) *Cg*1 and *Cg*2 are the centroids of the imidazolyl and C1–C6 benzene rings, respectively.

*D*—H⋯*A*	*D*—H	H⋯*A*	*D*⋯*A*	*D*—H⋯*A*
C12—H12*A*⋯*Cg*2^i^	0.93	2.80	3.364 (2)	120
C13—H13*A*⋯*Cg*1^i^	0.93	2.93	3.429 (5)	115

Symmetry code: (i) 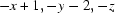.

**Fig. 1.**
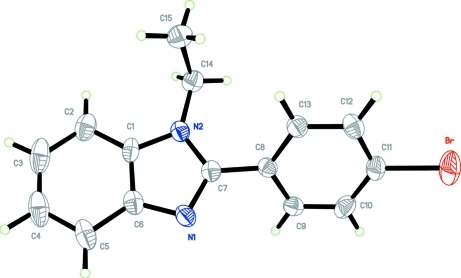
The molecular structure of title compound (I), with the atom-numbering scheme. Displacement ellipsoids are drawn at the 40% probability level.

**Fig. 2.**
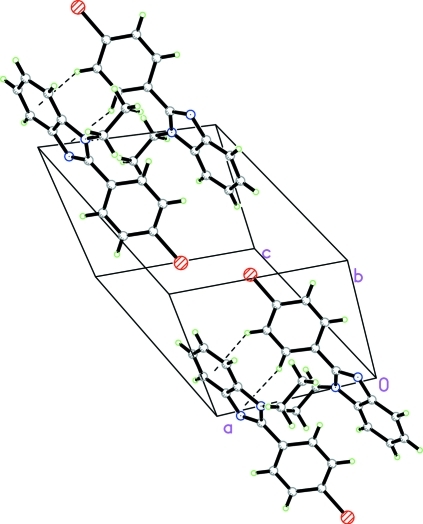
Packing diagram for (I) showing C—H···ring interactions.

## supplementary crystallographic information

### Comment

The tittle compound, (I), 2-(4-bromophenyl)-1-ethyl-1*H*-benzo[*d*]imidazole is an important intermediate organic intermediate which can be used for many fields such as OLED materials (Kakimoto *et al.*, 2008). Here we report the crystal structure of (I). The molecular structure of (I) is shown in Fig. 1, the bond lengths and angles are within normal ranges (Allen *et al.*, 1987).

In the benzimidazolyl group, the best planes through the phenyl ring and imidazolyl ring make an angle of 2.6 (2) °. Phenyl ring C8—C13 (connected with Br atom) makes an angle of 45.2 (3) ° and 43.3 (3) ° with phenyl ring C1—C6 and the imidazolyl ring, respectively.

In the crystal packing of (I), there were no classic intramolecular or intermolecular hydrogen bonds. Two C—H···ring interactions are present (Table 1, Fig.2).

### Experimental

The title compound, (I) was prepared by a method reported in literature (Kakimoto  *et al.*, 2008). The crystals were obtained by dissolving (I) (0.5 g, 1.61 mmol) in ethanol (25 ml) and evaporating the solvent slowly at room temperature for about 7 d.

### Refinement

All H atoms were positioned geometrically and constrained to ride on their parent atoms, with C—H = 0.93 Å for aromatic H, C—H = 0.96 Å and 0.97 Å for CH_2_ and CH_3_ groups, respectively, with *U*_iso_(H) = *xU*_eq_(C), where *x* = 1.2 for aromatic H, and *x* = 1.5 for other H atoms.

### Crystal data

**Table:**

C_15_H_13_BrN_2_	*Z* = 2
*M**_r_* = 301.18	*F*(000) = 304
Triclinic, *P*1	*D*_x_ = 1.533 Mg m^−^^3^
Hall symbol: -P 1	Mo *K*α radiation, λ = 0.71073 Å
*a* = 9.0780 (18) Å	Cell parameters from 25 reflections
*b* = 9.1480 (18) Å	θ = 10–14°
*c* = 9.2750 (19) Å	µ = 3.13 mm^−^^1^
α = 76.72 (3)°	*T* = 293 K
β = 78.44 (3)°	Block, colourless
γ = 61.05 (3)°	0.20 × 0.10 × 0.10 mm
*V* = 652.4 (2) Å^3^	

### Data collection

**Table:**

Enraf–Nonius CAD-4 diffractometer	1322 reflections with *I* > 2σ(*I*)
Radiation source: fine-focus sealed tube	*R*_int_ = 0.068
graphite	θ_max_ = 25.4°, θ_min_ = 2.3°
ω/2θ scans	*h* = 0→10
Absorption correction: ψ scan (North *et al.*, 1968)	*k* = −9→11
*T*_min_ = 0.573, *T*_max_ = 0.745	*l* = −10→11
2550 measured reflections	3 standard reflections every 200 reflections
2388 independent reflections	intensity decay: 1%

### Refinement

**Table:**

Refinement on *F*^2^	Primary atom site location: structure-invariant direct methods
Least-squares matrix: full	Secondary atom site location: difference Fourier map
*R*[*F*^2^ > 2σ(*F*^2^)] = 0.050	Hydrogen site location: inferred from neighbouring sites
*wR*(*F*^2^) = 0.111	H-atom parameters constrained
*S* = 1.00	*w* = 1/[σ^2^(*F*_o_^2^) + (0.049*P*)^2^] where *P* = (*F*_o_^2^ + 2*F*_c_^2^)/3
2388 reflections	(Δ/σ)_max_ < 0.001
163 parameters	Δρ_max_ = 0.28 e Å^−^^3^
0 restraints	Δρ_min_ = −0.32 e Å^−^^3^

### Special details

**Table:**

Geometry. All e.s.d.'s (except the e.s.d. in the dihedral angle between two l.s. planes) are estimated using the full covariance matrix. The cell e.s.d.'s are taken into account individually in the estimation of e.s.d.'s in distances, angles and torsion angles; correlations between e.s.d.'s in cell parameters are only used when they are defined by crystal symmetry. An approximate (isotropic) treatment of cell e.s.d.'s is used for estimating e.s.d.'s involving l.s. planes.
Refinement. Refinement of *F*^2^ against ALL reflections. The weighted *R*-factor *wR* and goodness of fit *S* are based on *F*^2^, conventional *R*-factors *R* are based on *F*, with *F* set to zero for negative *F*^2^. The threshold expression of *F*^2^ > σ(*F*^2^) is used only for calculating *R*-factors(gt) *etc*. and is not relevant to the choice of reflections for refinement. *R*-factors based on *F*^2^ are statistically about twice as large as those based on *F*, and *R*- factors based on ALL data will be even larger.

### Fractional atomic coordinates and isotropic or equivalent isotropic displacement parameters (Å^2^)

**Table:**

	*x*	*y*	*z*	*U*_iso_*/*U*_eq_	
Br	0.32320 (8)	−0.57697 (8)	0.48665 (6)	0.0929 (3)	
N1	0.1726 (4)	−0.8039 (4)	−0.1030 (4)	0.0524 (9)	
C1	0.2103 (5)	−1.0699 (6)	−0.0928 (5)	0.0519 (12)	
N2	0.2453 (4)	−1.0570 (5)	0.0412 (4)	0.0486 (9)	
C2	0.2144 (6)	−1.2057 (7)	−0.1407 (6)	0.0698 (14)	
H2A	0.2384	−1.3083	−0.0784	0.084*	
C3	0.1808 (7)	−1.1794 (9)	−0.2856 (7)	0.0872 (19)	
H3A	0.1828	−1.2662	−0.3234	0.105*	
C4	0.1437 (6)	−1.0237 (9)	−0.3760 (6)	0.0827 (18)	
H4A	0.1248	−1.0112	−0.4741	0.099*	
C5	0.1337 (5)	−0.8882 (7)	−0.3275 (5)	0.0694 (14)	
H5A	0.1054	−0.7847	−0.3892	0.083*	
C6	0.1682 (5)	−0.9129 (6)	−0.1804 (5)	0.0524 (12)	
C7	0.2206 (5)	−0.8950 (6)	0.0272 (5)	0.0461 (10)	
C8	0.2462 (5)	−0.8272 (5)	0.1432 (4)	0.0439 (10)	
C9	0.1320 (6)	−0.6632 (6)	0.1667 (5)	0.0538 (11)	
H9A	0.0382	−0.6028	0.1131	0.065*	
C10	0.1543 (6)	−0.5881 (6)	0.2670 (5)	0.0631 (13)	
H10A	0.0761	−0.4783	0.2818	0.076*	
C11	0.2940 (6)	−0.6771 (6)	0.3456 (5)	0.0552 (12)	
C12	0.4095 (6)	−0.8382 (6)	0.3237 (4)	0.0563 (12)	
H12A	0.5034	−0.8977	0.3773	0.068*	
C13	0.3864 (5)	−0.9121 (6)	0.2220 (4)	0.0515 (11)	
H13A	0.4665	−1.0209	0.2061	0.062*	
C14	0.2964 (6)	−1.1962 (6)	0.1673 (5)	0.0588 (12)	
H14A	0.2218	−1.2480	0.1842	0.071*	
H14B	0.2831	−1.1505	0.2564	0.071*	
C15	0.4772 (6)	−1.3301 (6)	0.1421 (5)	0.0673 (13)	
H15A	0.5041	−1.4173	0.2274	0.101*	
H15B	0.5519	−1.2802	0.1271	0.101*	
H15C	0.4905	−1.3781	0.0556	0.101*	

### Atomic displacement parameters (Å^2^)

**Table:**

	*U*^11^	*U*^22^	*U*^33^	*U*^12^	*U*^13^	*U*^23^
Br	0.1271 (6)	0.1207 (6)	0.0706 (4)	−0.0799 (5)	0.0003 (3)	−0.0424 (3)
N1	0.052 (2)	0.057 (2)	0.044 (2)	−0.018 (2)	−0.0118 (18)	−0.0083 (19)
C1	0.042 (3)	0.064 (3)	0.056 (3)	−0.022 (3)	0.000 (2)	−0.030 (3)
N2	0.046 (2)	0.055 (3)	0.047 (2)	−0.0224 (19)	−0.0062 (17)	−0.0112 (19)
C2	0.057 (3)	0.079 (4)	0.085 (4)	−0.033 (3)	0.001 (3)	−0.037 (3)
C3	0.069 (4)	0.128 (6)	0.098 (5)	−0.055 (4)	0.006 (3)	−0.068 (4)
C4	0.057 (4)	0.138 (6)	0.072 (4)	−0.045 (4)	−0.006 (3)	−0.051 (4)
C5	0.050 (3)	0.102 (4)	0.051 (3)	−0.024 (3)	−0.006 (2)	−0.028 (3)
C6	0.036 (3)	0.067 (4)	0.052 (3)	−0.019 (2)	−0.005 (2)	−0.019 (3)
C7	0.040 (3)	0.051 (3)	0.047 (3)	−0.020 (2)	0.000 (2)	−0.014 (2)
C8	0.045 (3)	0.048 (3)	0.037 (2)	−0.021 (2)	−0.002 (2)	−0.007 (2)
C9	0.057 (3)	0.048 (3)	0.051 (3)	−0.018 (3)	−0.013 (2)	−0.005 (2)
C10	0.075 (4)	0.048 (3)	0.066 (3)	−0.025 (3)	0.001 (3)	−0.022 (2)
C11	0.068 (3)	0.065 (3)	0.046 (3)	−0.039 (3)	0.001 (2)	−0.017 (2)
C12	0.050 (3)	0.072 (4)	0.047 (3)	−0.025 (3)	−0.005 (2)	−0.017 (2)
C13	0.045 (3)	0.052 (3)	0.053 (3)	−0.014 (2)	−0.006 (2)	−0.017 (2)
C14	0.065 (3)	0.057 (3)	0.058 (3)	−0.035 (3)	0.000 (2)	−0.007 (3)
C15	0.060 (3)	0.060 (3)	0.075 (3)	−0.023 (3)	−0.005 (3)	−0.012 (3)

### Geometric parameters (Å, °)

**Table:**

Br—C11	1.883 (4)	C8—C13	1.383 (5)
N1—C7	1.319 (5)	C8—C9	1.388 (6)
N1—C6	1.374 (5)	C9—C10	1.372 (6)
C1—N2	1.386 (5)	C9—H9A	0.9300
C1—C6	1.388 (6)	C10—C11	1.379 (6)
C1—C2	1.394 (6)	C10—H10A	0.9300
N2—C7	1.365 (5)	C11—C12	1.367 (6)
N2—C14	1.472 (5)	C12—C13	1.379 (5)
C2—C3	1.379 (7)	C12—H12A	0.9300
C2—H2A	0.9300	C13—H13A	0.9300
C3—C4	1.394 (8)	C14—C15	1.510 (6)
C3—H3A	0.9300	C14—H14A	0.9700
C4—C5	1.370 (7)	C14—H14B	0.9700
C4—H4A	0.9300	C15—H15A	0.9600
C5—C6	1.404 (6)	C15—H15B	0.9600
C5—H5A	0.9300	C15—H15C	0.9600
C7—C8	1.464 (5)		
			
C7—N1—C6	104.7 (4)	C10—C9—C8	121.6 (4)
N2—C1—C6	105.7 (4)	C10—C9—H9A	119.2
N2—C1—C2	130.8 (5)	C8—C9—H9A	119.2
C6—C1—C2	123.5 (4)	C9—C10—C11	119.2 (4)
C7—N2—C1	105.7 (4)	C9—C10—H10A	120.4
C7—N2—C14	130.3 (3)	C11—C10—H10A	120.4
C1—N2—C14	123.9 (4)	C12—C11—C10	120.5 (4)
C3—C2—C1	116.3 (5)	C12—C11—Br	119.9 (4)
C3—C2—H2A	121.9	C10—C11—Br	119.6 (4)
C1—C2—H2A	121.9	C11—C12—C13	119.7 (4)
C2—C3—C4	120.6 (5)	C11—C12—H12A	120.1
C2—C3—H3A	119.7	C13—C12—H12A	120.1
C4—C3—H3A	119.7	C12—C13—C8	121.1 (4)
C5—C4—C3	123.2 (5)	C12—C13—H13A	119.4
C5—C4—H4A	118.4	C8—C13—H13A	119.4
C3—C4—H4A	118.4	N2—C14—C15	112.8 (4)
C4—C5—C6	117.0 (5)	N2—C14—H14A	109.0
C4—C5—H5A	121.5	C15—C14—H14A	109.0
C6—C5—H5A	121.5	N2—C14—H14B	109.0
N1—C6—C1	110.4 (4)	C15—C14—H14B	109.0
N1—C6—C5	130.2 (5)	H14A—C14—H14B	107.8
C1—C6—C5	119.4 (4)	C14—C15—H15A	109.5
N1—C7—N2	113.5 (4)	C14—C15—H15B	109.5
N1—C7—C8	122.5 (4)	H15A—C15—H15B	109.5
N2—C7—C8	124.1 (4)	C14—C15—H15C	109.5
C13—C8—C9	117.8 (4)	H15A—C15—H15C	109.5
C13—C8—C7	123.5 (4)	H15B—C15—H15C	109.5
C9—C8—C7	118.5 (4)		
			
C6—C1—N2—C7	0.7 (4)	C14—N2—C7—N1	−179.2 (4)
C2—C1—N2—C7	−179.9 (4)	C1—N2—C7—C8	−179.0 (4)
C6—C1—N2—C14	−179.8 (4)	C14—N2—C7—C8	1.5 (7)
C2—C1—N2—C14	−0.3 (7)	N1—C7—C8—C13	−133.5 (4)
N2—C1—C2—C3	−176.6 (4)	N2—C7—C8—C13	45.7 (6)
C6—C1—C2—C3	2.7 (6)	N1—C7—C8—C9	40.5 (6)
C1—C2—C3—C4	−0.6 (7)	N2—C7—C8—C9	−140.3 (4)
C2—C3—C4—C5	−1.8 (8)	C13—C8—C9—C10	−1.5 (6)
C3—C4—C5—C6	2.0 (7)	C7—C8—C9—C10	−175.9 (4)
C7—N1—C6—C1	1.5 (5)	C8—C9—C10—C11	0.5 (7)
C7—N1—C6—C5	−176.5 (4)	C9—C10—C11—C12	0.2 (7)
N2—C1—C6—N1	−1.4 (5)	C9—C10—C11—Br	−178.6 (3)
C2—C1—C6—N1	179.1 (4)	C10—C11—C12—C13	0.1 (7)
N2—C1—C6—C5	176.9 (3)	Br—C11—C12—C13	179.0 (3)
C2—C1—C6—C5	−2.6 (6)	C11—C12—C13—C8	−1.2 (6)
C4—C5—C6—N1	178.1 (4)	C9—C8—C13—C12	1.9 (6)
C4—C5—C6—C1	0.2 (6)	C7—C8—C13—C12	175.9 (4)
C6—N1—C7—N2	−1.1 (5)	C7—N2—C14—C15	−106.0 (5)
C6—N1—C7—C8	178.2 (4)	C1—N2—C14—C15	74.6 (5)
C1—N2—C7—N1	0.2 (5)		

### Hydrogen-bond geometry (Å, °)

**Table:**

Cg1 and Cg2 are the centroids of the imidazolyl and C1–C6 benzene rings, respectively.

**Table:**

*D*—H···*A*	*D*—H	H···*A*	*D*···*A*	*D*—H···*A*
C12—H12A···Cg2^i^	0.93	2.80	3.364 (2)	120
C13—H13A···Cg1^i^	0.93	2.93	3.429 (5)	115

Symmetry codes: (i) −*x*+1, −*y*−2, −*z*.

## References

[bb1] Allen, F. H., Kennard, O., Watson, D. G., Brammer, L., Orpen, A. G. & Taylor, R. (1987). *J. Chem. Soc. Perkin Trans. 2*, pp. S1–19.

[bb2] Enraf–Nonius (1985). *CAD-4 Software* Enraf–Nonius, Delft, The Netherlands.

[bb3] Harms, K. & Wocadlo, S. (1995). *XCAD4* University of Marburg, Germany.

[bb4] Kakimoto, M., Ge, Z. Y., Hayakawa, T., Ando, S. & Ueda, M. (2008). *Adv. Funct. Mater.* A**18**, 584–590.

[bb5] North, A. C. T., Phillips, D. C. & Mathews, F. S. (1968). *Acta Cryst.* A**24**, 351–359.

[bb6] Sheldrick, G. M. (2008). *Acta Cryst.* A**64**, 112–122. 10.1107/S0108767307043930 18156677

